# Pd(OAc)_2_/Ph_3_P-catalyzed dimerization of isoprene and synthesis of monoterpenic heterocycles

**DOI:** 10.3762/bjoc.13.175

**Published:** 2017-08-29

**Authors:** Dominik Kellner, Maximilian Weger, Andrea Gini, Olga García Mancheño

**Affiliations:** 1 Institute of Organic Chemistry, University of Regensburg, Universitätsstr. 31, 93040 Regensburg, Germany; 2 Straubing Center of Science for Renewable Resources, 94315 Straubing, Germany

**Keywords:** dimerization, heterocycles, isoprene, monoterpene, palladium catalysis

## Abstract

The palladium-catalyzed dimerization of isoprene is a practical approach of synthesizing monoterpenes. Though several highly selective methods have been reported, most of them still required pressure or costly ligands for attaining the active system and desired selectivity. Herein, we present a simple and economical procedure towards the tail-to-tail dimer using readily available Pd(OAc)_2_ and inexpensive triphenylphosphine as ligand. Furthermore, simple screw cap vials are employed, allowing carrying out the reaction at low pressure. In addition, the potential of the dimer as a chemical platform for the preparation of heterocyclic terpenes by subsequent (hetero)-Diels–Alder or [4 + 1]-cycloadditions with nitrenes is also depicted.

## Introduction

The dimerization of conjugated dienes represents a useful, highly atom economic and straightforward reaction for the construction of important olefinic substituted synthetic building blocks [1–2]. Despite the high value of this type of compounds, there are only few robust methodologies that have led to commercial processes such as the Kuraray telomerization – dimerization with a nucleophile – of 1,3-butadiene with water followed by reduction to the linear 1-octanol [3–5], or the telomerization of 1,3-butadiene with methanol and reduction by the Dow Chemical Company to generate 1-octene [6–12]. Besides the well-studied case of 1,3-butadiene [5,13–15], only few reports for dimerization reactions of other 1,3-dienes are reported. In this regard, isoprene has recently attracted great attention as interesting branched C5-derivative for such dimerization processes. Isoprene is a broadly used, valuable chemical for the industry [16–17]. It is used for the synthesis of rubber and various other derivatives such as pheromones and fragrances. Though isoprene was initially obtained from thermal decomposition of natural rubber [18–19], nowadays it is mostly produced industrially as a byproduct of the cracking of naphtha or oil [20]. Recently, the bacterial production of bio-isoprene from renewable sources such as sugars has been considered as a competitive sustainable synthetic alternative [21–22].

**Figure 1:**
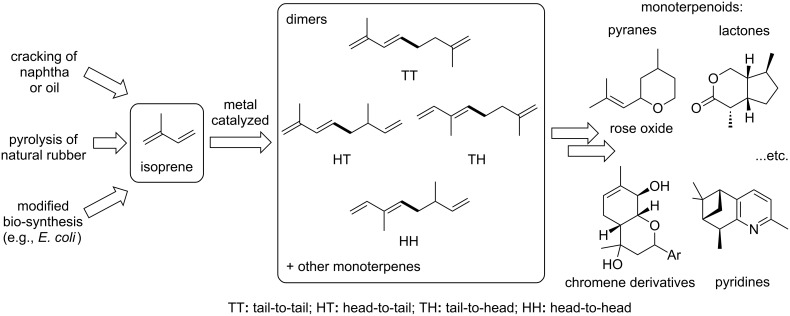
Isoprene as chemical building block in nature and organic synthesis.

Although the dimerization of isoprene is significantly more difficult compared to the dimerization of 1,3-butadiene, it would lead to synthetically useful linear monoterpenic structures that can be further transformed into interesting potential biologically active heterocyclic derivatives such as terpenoid pyranes, lactones, (hydro)chromenes or pyridines, among others [23–26]. Depending on the connection of the two isoprene units, four different kinds of dimers can be formed: tail-to-tail, head-to-tail, tail-to-head and head-to-head (Figure 1). In addition to the linear dimeric products, cyclic monoterpenes such as limonene can also be formed as byproducts.

**Scheme 1:**
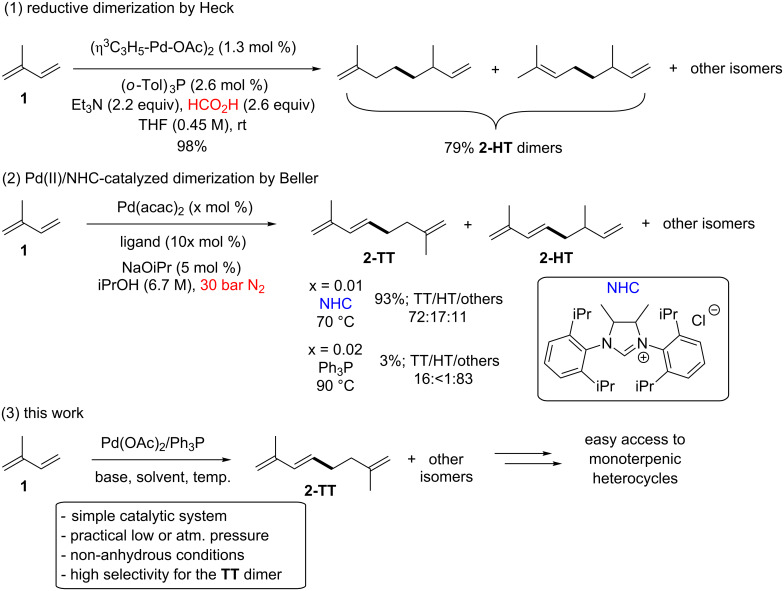
Pd-catalyzed dimerization of isoprene.

A number of transition metal complexes can be used as catalysts in the dimerization of isoprene. In one hand, titanium-, zirconium-, iron-, cobalt-, vanadium- or aluminum-based Ziegler-type catalysts lead mostly to the linear tail-to-head **2-TH** dimer 2,6-dimethyl-1,3,6-octatriene (alloocimene) [27], and the use of nickel catalysts allows the preparation of the tail-to-tail dimer **2-TT** (2,7-dimethyl-1,3,7-octatriene) [28]. On the other hand, palladium appears to be the most versatile metal for such a dimerization reaction, since the right choice of the appropriate catalytic system permits the control of the selectivity of the process. Thus, several catalysts such as Pd(Ph_3_P)_4_ or Pd(Ph_3_P)_2_(maleic anhydride) favor the tail-to-tail linkage [15,29]. Moreover, the naturally occurring head-to-tail **2-HT** dimers, myrcene and ocimene, can be obtained in allylic alcohol when employing Pd(NO_3_)_2_/Ph_3_P/KOPh as catalytic system [30]. The head-to-head **2-HH** dimer could also be prepared as major isomer under catalysis with the system PdBr_2_(dppe)/NaOPh/PhOH [31]. Furthermore, an interesting work by Heck and co-workers in 1976 already showed the possibility of generating the corresponding dimethyloctadienes of isoprene by a reductive dimerization in the presence of formic acid with the dimeric allylpalladium acetate catalyst and a bulky monodentated phosphine (Scheme 1, reaction 1) [32]. More recently, important efforts have been made in order to develop new methodologies towards more effective and selective catalytic systems, such as the introduction of highly active *N*-heterocyclic carbene (NHC) catalysts reported by Beller and co-workers (Scheme 1, reaction 2) [33]. The use of NHCs proved to be the key, since the more accessible monodentated phosphorus ligands such as triphenyl (Ph_3_P) or tricyclohexyl (Cy_3_P) phosphines showed a significant lower efficiency and different selectivity with the same palladium source Pd(acac)_2_. However, and despite the good selectivity obtained towards the tail-to-tail dimer **2-TT**, this Pd/NHC catalysis required to be applied under pressure conditions (30 or 50 bar) for attaining the reported performance. Therefore, it would be highly desirable to develop simple, more readily available and general catalytic methods for such transformation, as well as the functionalization of the dimers to more complex derivatives.

Herein, we present our work towards the dimerization of isoprene and the modification of its dimers into valuable heterocyclic products with potential commercial applications such as pesticides, odors or flavors (Scheme 1, reaction 3).

## Results and Discussion

### Dimerization of isoprene

We focused our study on the use of commercially available palladium catalysts and inexpensive Ph_3_P as catalytic system under practical non-anhydrous and atmospheric or low-pressure conditions for the formation of the corresponding isoprene dimers **2**. Therefore, the reaction conditions for the Pd-catalyzed dimerization of isoprene were first shortly optimized (Table 1). The ratio of the products in the resulting mixtures was analyzed by NMR spectroscopy and GC spectrometry.

**Table 1:** Optimization of the dimerization reaction of isoprene.^a^

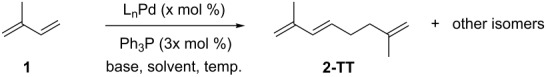							

Entry	catatyst (mol %)	Ph_3_P (mol %)	Base	solvent	*T* (°C)	Selectivity^b^ **2-TT**:others	Yield **2-TT** (%)^c^ (**2** total %)^d^

1	Pd(OAc)_2_ (5)	–	Et_3_N	THF	90	1:9	<2 (15)
2	Pd(Ph_3_P)_2_Cl_2_ (2)	–	Et_3_N	THF	90	–	traces
3	Pd(Ph_3_P)_4_ (2)	–	Et_3_N	THF	90	–	traces
4	Pd(OAc)_2_ (2)	(6)	Et_3_N	THF	90	16:1	86 (91)
5	Pd(OAc)_2_ (2)	(6)	Et_3_N	THF	70	–	traces
6	Pd(OAc)_2_ (2)	(6)	Et_3_N	THF	80	–	traces
7^e^	Pd(OAc)_2_ (2)	(6)	Et_3_N	THF	120	2:1	46 (70)
8	Pd(OAc)_2_ (1)	(3)	Et_3_N	THF	90	16:1	85 (90)
9^f^	Pd(OAc)_2_ (1)	(3)	Et_3_N	THF	90	10:1	80 (87)
10	Pd(OAc)_2_ (1)	(3)	KOH	THF	90	–	traces
11	Pd(OAc)_2_ (1)	(3)	K_2_CO_3_	THF	90	4:3	5 (9)
12	Pd(OAc)_2_ (1)	(3)	KHCO_3_	THF	90	4:1	14 (17)
13^g^	Pd(OAc)_2_ (1)	(3)	KHCO_3_	THF	70	19:1	25 (27)
14	Pd(OAc)_2_ (1)	(3)	KHCO_3_	MeOH	90	1:8	6 (46)^h^
15	Pd(OAc)_2_ (1)	(3)	Et_3_N	EtOAc	90	9:1	27 (30)
16	Pd(OAc)_2_ (1)	(3)	Et_3_N	toluene	90	7:1	78 (89)
17	Pd(OAc)_2_ (0.5)	(1.5)	Et_3_N	THF	90	15:1	70 (74)
18^g^	Pd(OAc)_2_ (0.02)	(0.06)	Et_3_N	THF	90	–	traces^i^

^a^Reaction conditions: Isoprene (1 mL, 10 mmol, 2 equiv), Pd-catalyst (x mol %), Ph_3_P ligand (3x mol %, when required), base (10 mol %) and solvent (2 mL, 5 M) at the corresponding temperature for 48 h. ^b^Selectivity determined by NMR and GC–FID. ^c^Yield of **2-TT** determined by GC–FID using decanol as internal standard. ^d^Total yield of monoterpenes in brackets. ^e^12 hours of reaction time. ^f^Use of 40 mol % of Et_3_N as base. ^g^4 days of reaction time. ^h^The formation of a mixture of methanol-telomers was observed. ^i^The major formation of cyclic monoterpenes (7%) was observed.

The reaction using 2 mol % of Pd(OAc)_2_, Pd(Ph_3_P)_2_Cl_2_ or Pd(Ph_3_P)_4_ as catalyst in the presence of 10 mol % Et_3_N as base in THF at 90 °C in a sealed Schlenk tube was initially explored. However, a low conversion into the tail-to-tail **2-TT** (2% yield for Pd(OAc)_2_) or only traces (for the other two catalysts) of the desired linear dimers **2** could be detected (Table 1, entries 1–3). Based on the previous reported work on the dimerization of 1,3-butadiene and isoprene, in which monodentated ligands were employed, the Pd(OAc)_2_/Ph_3_P/Et_3_N (1:3:10) catalytic system was next explored. This combination led to a significant increase of the reactivity (86% yield), delivering the **TT**-linear terpene as the main product in a 16:1 ratio of **2-TT** vs other dimeric isomers **2**, along with Diels–Alder-generated cyclic monoterpene byproducts (Table 1, entry 4). Interestingly, the dimerization did not take place at temperatures below 90 °C (Table 1, entries 5 and 6). Increasing the temperature to 120 °C was translated to a faster reaction, but a significant decrease on the selectivity was observed (Table 1, entry 7). Therefore, 90 °C was chosen as the reaction temperature for the further optimization studies.

Next, the effect of the base in the outcome of the reaction was explored. In order to attain higher conversion into the dimers **2**, the amount of base was increased to 40% (Table 1, entry 9). However, it led to a notably decrease of the yield and selectivity. Furthermore, different inorganic bases such as KOH, K_2_CO_3_ or KHCO_3_ were then investigated (Table 1, entries 10–14). This change of the base was not translated to any significant improvement. However, it is worth to mention that in the case of KHCO_3_ it was possible to form the dimer **2-TT** in a moderate 25% yield and an improved 20:1 selectivity at a temperature of 70 °C (Table 1, entry 13), in which almost no reactivity was observed with triethylamine as base (Table 1, entry 5).

The effect of the solvent was then evaluated (Table 1, entries 14–16). Besides THF, ethyl acetate, toluene and methanol were tested. However, all these solvents resulted in lower yields and selectivities towards **2-TT**. Therefore, THF was identified as optimal solvent. Furthermore, when using methanol as solvent in the presence of KHCO_3_, significant amounts of byproducts were observed (Table 1, entry 14). In this case, a methanol molecule was added to the dimeric structure, forming as expected all possible different MeOH-telomers in 3–5% of the yield.

Finally, the catalyst loading could be decreased to 0.5 mol %, maintaining good levels of reactivity (70%) and without observing any loss of selectivity (15:1 **2-TT**/others, Table 1, entry 17). However, it was not possible to reduce further the catalyst amount to 0.02 mol % of Pd(OAc)_2_, since only traces of the desired dimers **2** were obtained (Table 1, entry 18). Under these conditions, the main products detected were cyclic monoterpenes, which were only obtained in a low 7% yield.

**Scheme 2:**
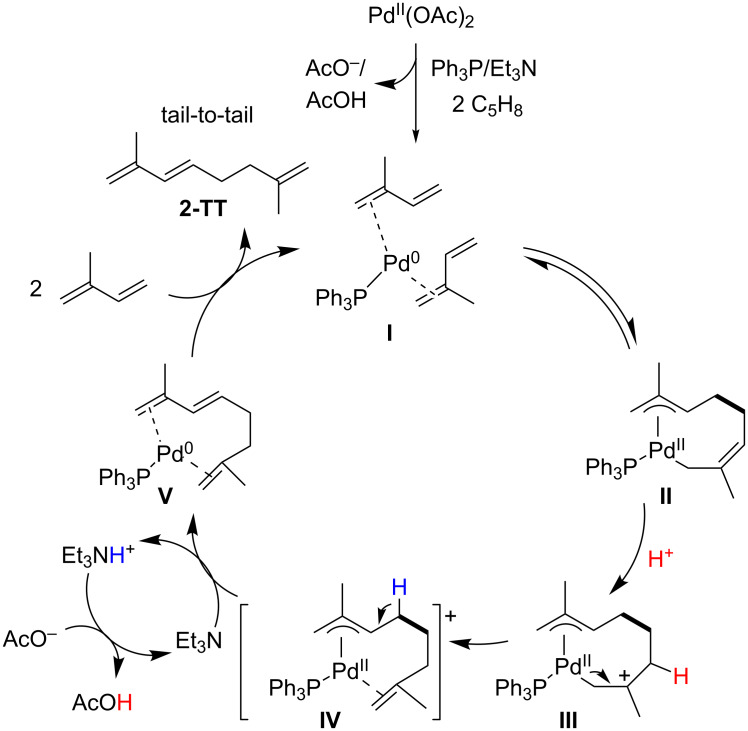
Putative mechanism for the Pd(OAc)_2_-catalyzed dimerization of isoprene.

After having identified the optimal reaction conditions for the dimerization reaction implying the Pd(OAc)_2_/Ph_3_P/Et_3_N-system, a plausible catalytic cycle is suggested based on our observations and previous Pd-catalyzed reports [34–37]. Thus, the mechanism for dimerization to the major tail-to-tail dimer **2-TT** is shown in Scheme 2. First of all, the Pd(OAc)_2_ is getting reduced in situ in the presence of Et_3_N and Ph_3_P to Pd(0) [13,20], which is the reactive species for the dimerization. Then, two molecules of isoprene coordinate to the Pd-metal center (species **I**). Upon the C–C coupling between the two isoprene units, a Pd-(η^1^,η^3^-dimethyloctadiendiyl)-complex of type **II** (as exemplified for the tail-to-tail attack) is formed. Next, the protonation of **II** with the in situ formed catalytic acetic acid occurs to generate the cationic intermediate **III**, which further evolves to the π-allyl–Pd(II) complex **IV**. Then, the formation of the π–π-Pd(0) complex **V** takes place by a base-catalyzed β-hydrogen elimination. Finally, the isoprene dimer **2-TT** is liberated by complexation of the Pd(0) with two other molecules of isoprene, regenerating the complex **I** that enters in the next catalytic cycle.

### Functionalization of the tail-to-tail dimer: Synthesis of monoterpenic *O*- and *N*-heterocycles

**Scheme 3:**
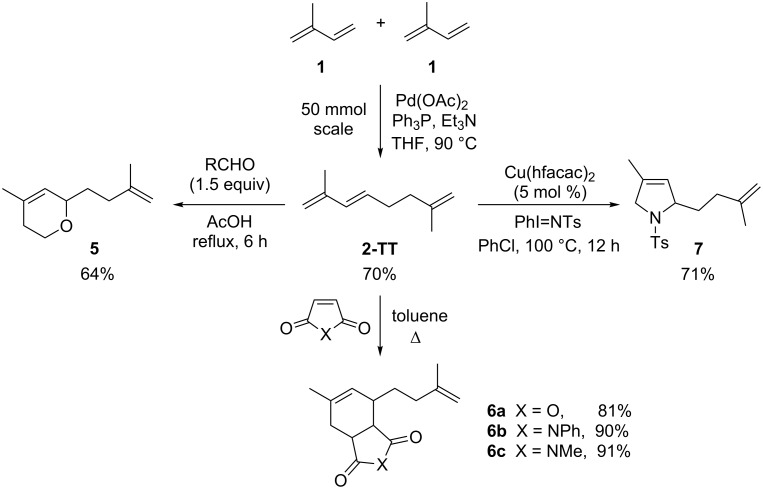
Functionalization of the isoprene-dimer **2-TT** to substituted *O*- and *N*-heterocycles.

The different dimers of isoprene can be transformed into a large number of derivatives such as terpene-alcohols or ethers [38]. Moreover, some isoprene dimers have been submitted to Diels–Alder reactions with olefins such as maleic acid anhydride or methacrolein to form products that are used as alkyd coatings and perfume additives [39–40]. In addition, further functionalization reactions of the isoprene dimers can be carried out. In this work, we envisioned the synthesis of various heterocycles by (hetero)Diels–Alder and [4 + 1]-cycloadditions with nitrenes with the obtained major tail-to-tail dimer **2-TT** (Scheme 3).

Initially, the dimerization reaction was scaled-up to 50 mmol under the optimized conditions using 1 mol % of Pd(OAc)_2_ (Table 1, entry 8), providing the **2-TT** dimer in 70% yield after fractional distillation (≈90% purity) [41]. In a first instance, the synthesis of pyranes by a hetero-Diels–Alder reaction was realized. Thus, the reaction of **2-TT** with formaldehyde in acetic acid at reflux led after 6 h exclusively to one regioisomer dihydropyrane **5** in 64% (Scheme 3, left) [42]. Next, the thermal Diels–Alder reaction with maleic anhydride and *N*-substituted maleimides in toluene provided a ready access to the fused ring-system **6a–c** in excellent yields (81–91%; Scheme 3, middle).

Finally, the [4 + 1]-cycloaddition with nitrenes was carried out (Scheme 3, right). To attain this transformation the required nitrene was formed in situ from an iminoiodinane precursor (PhI=NR) under metal catalysis [43–46]. Although the formal [4 + 1]-cycloaddition with 1,3-dienes has been already described [47], isoprene dimers have not been enrolled as substrates in such type of reaction since the presence of the additional, less hindered double bond could lead to undesired competitive side-reactions such as the aziridination at this position [48–49]. However, the desired substituted 3-pyrroline derivatives of type **7** will permit additional functionalization, offering many synthetic possibilities. Encouraged by this, we employed the Cu(hfacac)_2_ catalyts as reported for alkyl and aryl mono-, bi- and tri-substituted 1,3-dienes with *N*-(*p*-tolylsulfonyl)imino]phenyliodinane (PhI=NTs) [37]. To our delight, the desired 3-pyrroline **7** was obtained as major product in a good 71% yield.

## Conclusion

In summary, a simple and practical Pd-catalyzed dimerization of isoprene has been developed. A high selectivity towards the tail-to-tail dimer was achieved by using readily available Pd(OAc)_2_ and inexpensive triphenylphosphine as ligand in the presence of catalytic amounts of Et_3_N as base. The reaction can be performed with common chemicals and solvents at low pressure in simple screw cap vials or schlenks, leading to the desired dimer in good yields. Finally, the synthetic potential of this method for the synthesis of valuable heterocyclic terpenic derivatives was demonstrated with the preparation of several pyran, tetrahydroisobenzofurane and pyrrolidine derivatives by subsequent cyclization involving (hetero)-Diels–Alder or [4 + 1]-cycloaddition reactions.

## Experimental

### General Information

^1^H and ^13^C NMR spectra were recorded in CDCl_3_ (reference signals [50]: ^1^H = 7.26 ppm, ^13^C = 77.16 ppm) on a 400 MHz Jeol spectrometer. Chemical shifts (δ) are given in ppm and spin–spin coupling constants (J) are given in Hz. Analytical thin layer chromatography was performed using silica gel 60 F254 and column chromatography was performed on silica gel 60 (0.040–0.063 mm). The chemical yields and product distributions were determined or monitorized by GC on a Shimadzu GC-FID “GC-2025 AF” and a GC–MS “QP-2010 SE”. Exact masses (HRMS) were recorded on an Agilent Q-TOF 6540 UHD spectrometer. Commercially available reagents were used without further purification.

### Pd-catalyzed dimerization of isoprene

The reaction was performed under nitrogen in a screw-cap vial. Pd(OAc)_2_ (11.2 mg, 0.05 mmol, 1 mol %), Ph_3_P (39.3 mg, 0.15 mmol, 3 mol %) and isoprene (**1**, 1 mL, 10 mmol, 2 equiv) were dissolved in THF (5 M). Subsequently, Et_3_N (69 μL, 0.5 mmol, 10 mol %) was added and the reaction mixture was stirred at 90 °C for 48 hours. After purification of the reaction mixture by column chromatography (gradient: hexane to hex/EtOAc 4:1), the desired dimer **2-TT** (578 mg, 4.25 mmol, 85%) was obtained as colorless oil and the major product (16:1, **2-TT**/others). The reaction on 50 mmol scale was purified by fractional distillation (70 °C, ≈6 mbar; 70% **2-TT**).

#### (*E*)-2,7-Dimethylocta-1,3,7-triene (**2-TT**) [33]


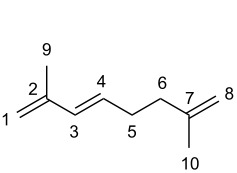


^1^H NMR (400 MHz, CDCl_3_) δ 6.17 (d, *J* = 15.5 Hz, 1H, H-3), 5.67 (dt, *J* = 15.5, 7.0 Hz, 1H, H-4), 4.88 (s, 2H, H-1), 4.72 (bd, *J* = 12.0 Hz, 2H, H-8), 2.31–2.22 (m, 2H, H-6), 2.17–2.06 (m, 2H, H-5), 1.84 (s, 3H, Me-9), 1.74 (s, 3H, Me-10); ^13^C NMR (100 MHz, CDCl_3_) δ 145.3 (C-7), 142.1 (C-2), 133.0 (C-3), 130.2 (C-4), 114.3 (C-1), 110.1 (C-8), 37.6 (C-6), 31.0 (C-5), 22.4 (Me-10), 18.6 (Me-9); MS (EI^+^) *m*/*z*: 136 [M^+.^] (4), 121 [M − Me]^+^ (36), [C_8_H_11_]^+^ 107 (25), 93 (22), [C_6_H_10_]^+^ 82 (11), [C_6_H_9_]^+^ 81 (100), [C_5_H_7_]^+^ 79 (87), [C_4_H_7_]^+^ 55 (21), 53 (42).

#### Functionalization of the isoprene-dimer **2-TT**

**Hetero Diels–Alder reaction:** The dimer **2-TT** (136.0 mL, 1.0 mmol, 1 equiv) was dissolved in acetic acid (5 mL, 0.2 M) and paraformaldehyde (45.0 mg, 1.5 mmol, 1.5 equiv) was added. The mixture was stirred and heated under reflux for 3–6 hours until completion, which was monitored by TLC. Then, the mixture was cooled down to room temperature, poured into an ice-cold saturated Na_2_CO_3_ solution and extracted three times with diethyl ether. The organic phase was separated, washed with distilled water and dried over MgSO_4_. The purification of the reaction mixture was carried out by column chromatography (gradient: hexane to hexanes/EtOAc 4:1) and distillation (90 °C, ≈6 mbar), to provide pyran **5** as colorless oil (106.0 mg, 0.64 mmol, 64%).

#### 4-Methyl-2-(3-methylbut-3-en-1-yl)-5,6-dihydro-*2H*-pyran (**5**) [42]


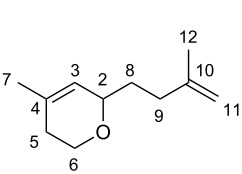


^1^H NMR (400 MHz, CDCl_3_) δ 5.37–5.31 (m, 1H, H-2), 4.80–4.63 (m, 2H, H-11), 4.04–3.90 (m, 2H, H-2 + H-6a), 3.61 (m, 1H, H-6b), 2.30–1.99 (m, 3H, H-9 + H-5a), 1.84–1.75 (m, 1H, H-5b), 1.73 (s, 3H, Me-7), 1.70 (s, 3H, Me-12), 1.66–1.58 (m, 2H, H-8); ^13^C NMR (100 MHz, CDCl_3_) δ 145.9 (C-10), 132.4 (C-4), 123.9 (C-3), 109.7 (C-11), 73.6 (C-2), 63.5 (C-6), 33.7 (C-8), 33.4 (C-9), 30.1 (C-5), 23.2 (Me-7), 22.6 (Me-12); MS (EI^+^) *m*/*z*: 166 [M^+.^] (1), 110 [C_7_H_10_O]^+^ (93), 97 [C_6_H_9_O]^+^ (100), 79 [C_6_H_7_]^+^ (19), 69 [C_5_H_9_]^+^ (26), 67 (16), 55 (19).

#### Diels–Alder reactions

**General procedure:** Dimer **2-TT** (1.5 equiv) and the corresponding dienophile (1 equiv) were dissolved in toluene (0.5 M) and stirred for 4 hours at 150 °C. The solvent of the reaction mixture was evaporated and the residue put under high vacuum overnight, leading to the desired pure Diels–Alder products.

#### 6-Methyl-4-(3-methylbut-3-en-1-yl)-3a,4,7,7a-tetrahydroisobenzofuran-1,3-dione (**6a**)


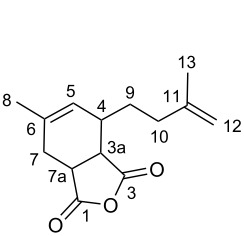


Following the general procedure, the reaction of **2-TT** with maleic anhydride (74 mg, 1.0 mmol, 1.0 equiv) gave **6a** as a colorless oil (190 mg, 0.81 mmol, 81%). ^1^H NMR (400 MHz, CDCl_3_) δ 5.48 (bs, 1H, H-5), 4.75 (bs, 1H, H-12a), 4.72 (bs, 1H, H-12b), 3.42 (ddd, *J* = 9.6, 7.0, 2.4 Hz, 1 H, H-7a), 3.32 (dd, *J* = 9.6, 6.5 Hz, 1H, H-3a), 2.57 (dd, *J* = 15.2, 1.9 Hz, 1H, H-4), 2.28–2.20 (m, 2H, H-10), 2.19–2.16 (m, 2H, H-7), 1.99–1.90 (m, 1H, H-9a), 1.87–1.79 (m, 1H, H-9b), 1.78 (s, 3H, Me-13), 1.75 (s, 3H, Me-8); ^13^C NMR (100 MHz, CDCl_3_) δ 174.3 (C-1), 171.6 (C-3), 144.8 (C-7), 137.2 (C-12), 126.3 (C-6), 110.8 (C-13), 43.6 (C-4), 41.4 (C-9), 35.7 (C-11), 35.4 (C-8), 29.3 (C-5), 28.8 (C-10), 23.2 (C-15), 22.2 (C-14); MS (EI^+^) *m*/*z*: 234 [M^+.^] (3), 178 [M − C_4_H_8_]^+^ (10), 150 (18), 107 [C_8_H_11_]^+^ (10), 106 (47), 105 (37), 93 (59), 91 (100), 79 [C_6_H_7_]^+^ (28), 77 (58), 69 [C_5_H_9_]^+^ (81), 55 [C_4_H_7_]^+^ (25).

#### 6-Methyl-4-(3-methylbut-3-en-1-yl)-2-phenyl-3a,4,7,7a-tetrahydro-*1H*-isoindole-1,3(2*H*)-dione (**6b**)


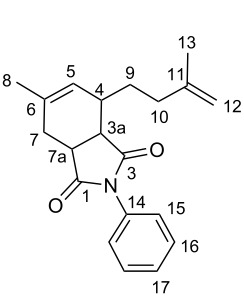


Following the general procedure, the reaction of **2-TT** with *N*-phenylmaleimide (173 mg, 1.0 mmol, 1.0 equiv) gave **6b** as a pale yellow oil (278 mg, 0.9 mmol, 90%). ^1^H NMR (400 MHz, CDCl_3_) δ 7.44 (t, *J* = 7.8 Hz, 2H, H-15), 7.36 (t, *J* = 7.5 Hz, 1H, H-17), 7.18 (d, *J* = 8.0 Hz, 2H, H-16), 5.48 (bs, 1H, H-5), 4.73 (bs, 2H, H-12), 3.28 (ddd, *J* = 8.8, 6.9, 1.7 Hz, 1H, H-7a), 3.23 (dd, *J* = 8.8, 6.1 Hz, 1H, H-3a), 2.35–2.25 (m, 2H, H-10), 2.21 (bt, *J* = 7.6 Hz, 1H, H-4), 2.12–2.01 (m, 1H, H-7), 1.90–1.80 (m, 1H, H-9), 1.80 (s, 3H, H-13), 1.76 (s, 3H, H-8); ^13^C NMR (100 MHz, CDCl_3_) δ 179.1 (C-1), 177.2 (C-3), 145.5 (C-6), 136.6 (C-11), 132.1 (C-14), 129.2 (C-16), 128.6 (C-17), 126.6 (C-15), 126.3 (C-5), 110.5 (C-12), 43.1 (C-7a), 40.8 (C-3a), 36.5 (C-10), 36.2 (C-7), 29.7 (C-4), 29.2 (C-9), 23.2 (C-8), 22.4 (C-13); MS (EI^+^) *m*/*z*: 309 [M^+.^] (3), 253 [M − C_4_H_8_]^+^ (39), 241 [C_6_H_9_O]^+^ (14), 106 (100), 93 (65), 91 (89), 77 (67), 69 (30), 56 (28), 55 (28).

#### 2,6-Dimethyl-4-(3-methylbut-3-en-1-yl)-3a,4,7,7a-tetrahydro-*1H*-isoindole-1,3(2*H*)-dione (**6c**)


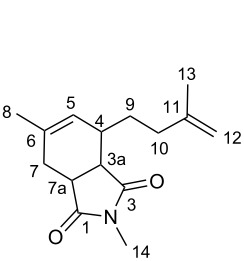


Following the general procedure, the reaction of **2-TT** with *N*-methylmaleimide (111 mg, 1.0 mmol, 1.0 equiv) gave **6c** as a colorless oil (225 mg, 0.91 mmol, 91%). ^1^H NMR (400 MHz, CDCl_3_) δ 5.35 (bs, 1H, H-5), 4.74–4.70 (m, 2H, H-14), 3.12–3.00 (m, 2H, H-3a + H-7a), 2.89 (s, 3H, Me-14), 2.53 (dd, *J* = 14.9, 1.6 Hz, 1H, H-4), 2.26–2.10 (m, 4H, H-7 + H-9), 2.05–1.95 (m, 1H, H-9a), 1.79 (ddd, J = 14.3, 8.5, 6.6 Hz, 1H, H-9b), 1.74 (s, 3H, Me-13), 1.70 (s, 3H, Me-8); ^13^C NMR (100 MHz, CDCl_3_) δ 179.9 (C-1), 178.2 (C-3), 145.4 (C-6), 136.3 (C-11), 126.0 (C-5), 110.4 (C-12), 42.9 (C-7a), 40.7 (C-3a), 36.1 (C-10), 36.0 (C-7), 29.3 (C-4), 29.2 (C-9), 24.7 (C-14), 23.1 (C-8), 22.3 (C-13); MS (EI^+^) *m*/*z*: 247 [M^+.^] (3), 191 (80), 179 (36), 178 [M-C_5_H_9_]^+^ (13), 112 (25), 107 (26), 106 [C_6_H_7_]^+^ (100), 93 (81), 91 (86), 79 (37), 77 (59), 69 (60), 55 [C_4_H_7_]^+^ (42), 53 (37).

#### Formal [4 + 1]-cycloaddition [47]

Catalyst Cu(hfacac)_2_ (18 mg, 0.04 mmol, 5 mol %) and the nitrene precursor PhI=NTs (280 mg, 0.75 mmol, 1.0 equiv) were dissolved in chlorobenzene (5 mL, 0.15 M). To this solution, the synthesized dimer **2-TT** (136 mg, 1.00 mmol, 1.3 equiv) was added. The reaction mixture was stirred at room temperature for 1 h and further heated at 100 °C for 24 h. After purification of the crude mixture by column chromatography (gradient: hexanes to hexanes/EtOAc 9:1 to 4:1), pyrrole derivative **7** was obtained as a colorless solid (162 mg, 0.53 mmol, 71%).

#### 4-Methyl-2-(3-methylbut-3-en-1-yl)-1-tosyl-2,5-dihydro-*1H*-pyrrole (**7**)


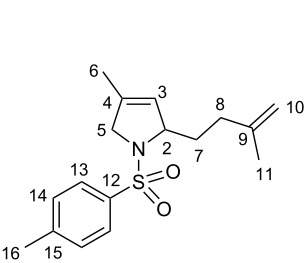


^1^H NMR (400 MHz, CDCl_3_) δ 7.69 (d, *J* = 8.3 Hz, 2H, H-13), 7.28 (d, *J* = 8.3 Hz, 2H, H-14), 5.19 (dt, *J* = 3.3, 1.6 Hz, 1H, H-3), 4.72–4.65 (m, 2H, H-10), 4.47–4.43 (m, 1H, H-2), 4.04–3.88 (m, 2H, H-5), 2.40 (s, 3H, Me-16), 2.11–1.79 (m, 4H, H-7 + H-8), 1.72 (s, 3H, Me-11), 1.62 (s, 3H, Me-6); ^13^C NMR (100 MHz, CDCl_3_) δ 145.4 (C-9), 143.2 (C-12), 134.8 (C-15), 134.4 (C-4), 129.6 (C-14), 127.3 (C-13), 123.3 (C-3), 109.9 (C-10), 67.2 (C-2), 58.5 (C-5), 34.1 (C-7), 32.7 (C-8), 22.4 (Me-6), 21.4 (Me-16), 13.8 (Me-10); HRMS (ESI^+^): calcd. for [C_17_H_24_NO_2_S]^+^: 306.1528; found: 306.1529.

## Supporting Information

File 1 ^1^H NMR and ^13^C NMR spectra collection of the products and GC–FID analysis of the isoprene dimer’s mixture.
