# Supplementary material for: Pd(OAc)2/Ph3P-catalyzed dimerization of isoprene and synthesis of monoterpenic heterocycles
Source: Beilstein J Org Chem. 2017 Aug 29;13:1807–15. doi: 10.3762/bjoc.13.175 (PMC5588454; doi:10.3762/bjoc.13.175)

Supporting Information

for

$Pd(OAc)_2/Ph_3P$-catalyzed dimerization of isoprene and synthesis of monoterpenic heterocycles

Dominik Kellner[1,2], Maximilian Weger[1,2], Andrea Gini[1,2] and Olga García Mancheño*[1,2]

Address: [1]Institute of Organic Chemistry, University of Regensburg, Universitätsstr. 31, 93040 Regensburg, Germany and [2]Straubing Center of Science for Renewable Resources, 94315 Straubing, Germany

Email: Olga García Mancheño* - olga.garcia-mancheno@ur.de

*Corresponding author

^1H NMR and ^{13}C NMR spectra collection of the products and GC–FID analysis of the isoprene dimer's mixture

Table of contents

1. NMR spectra
2. GC–FID Analysis

1. NMR spectra

Isoprene dimer:

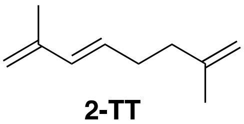

2-TT

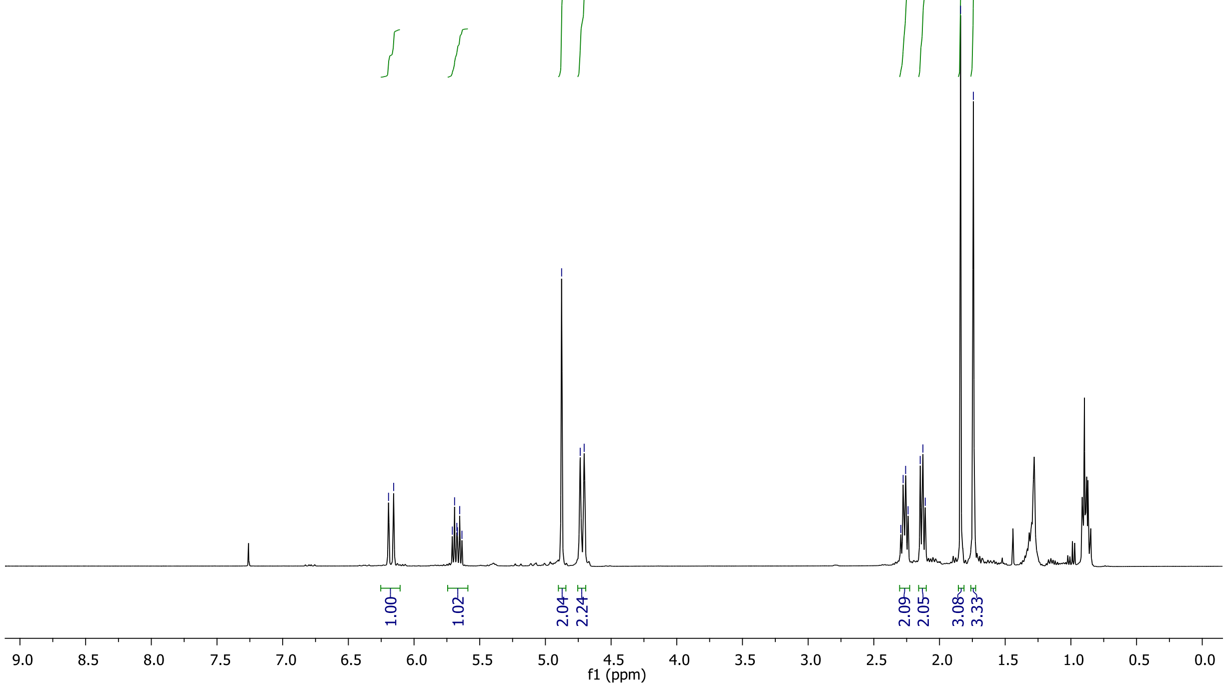

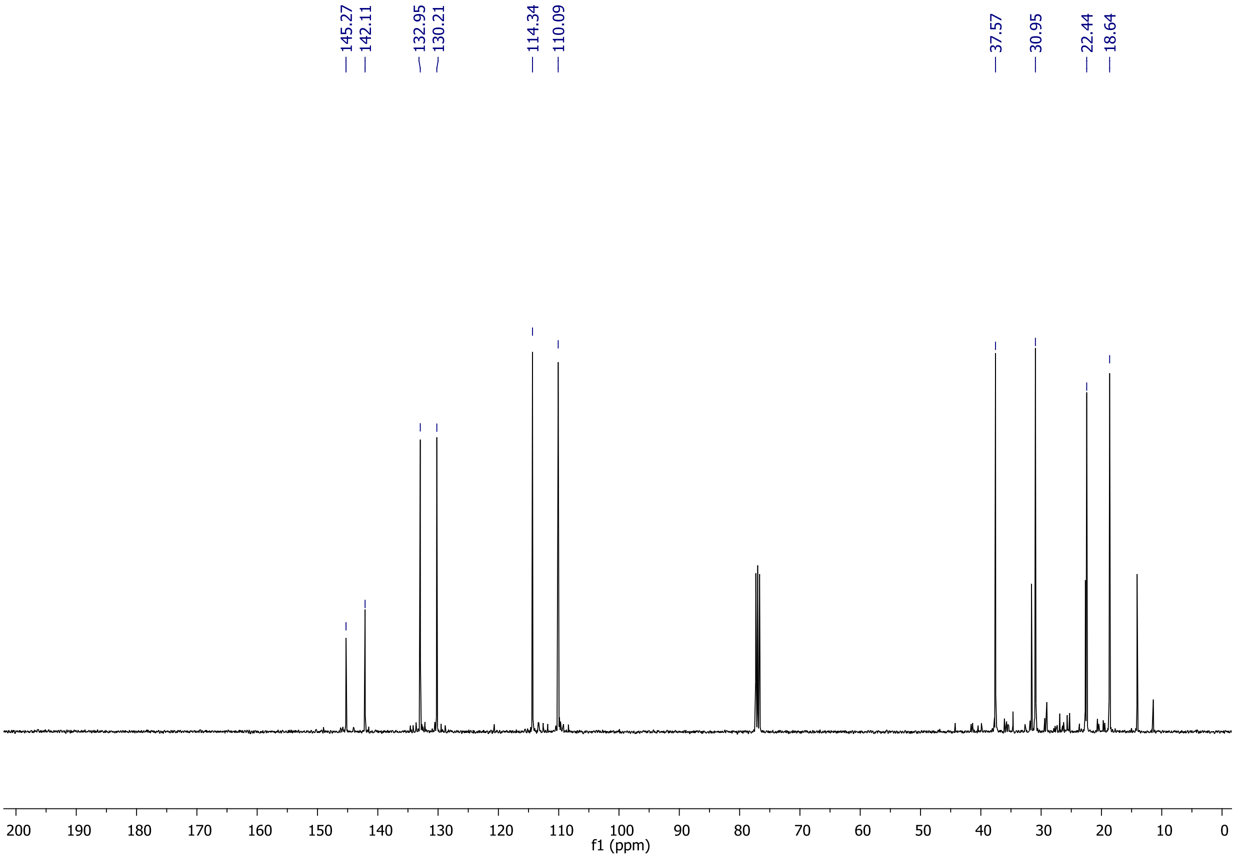

Pyrane:

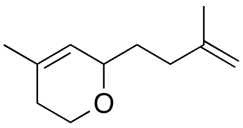

5

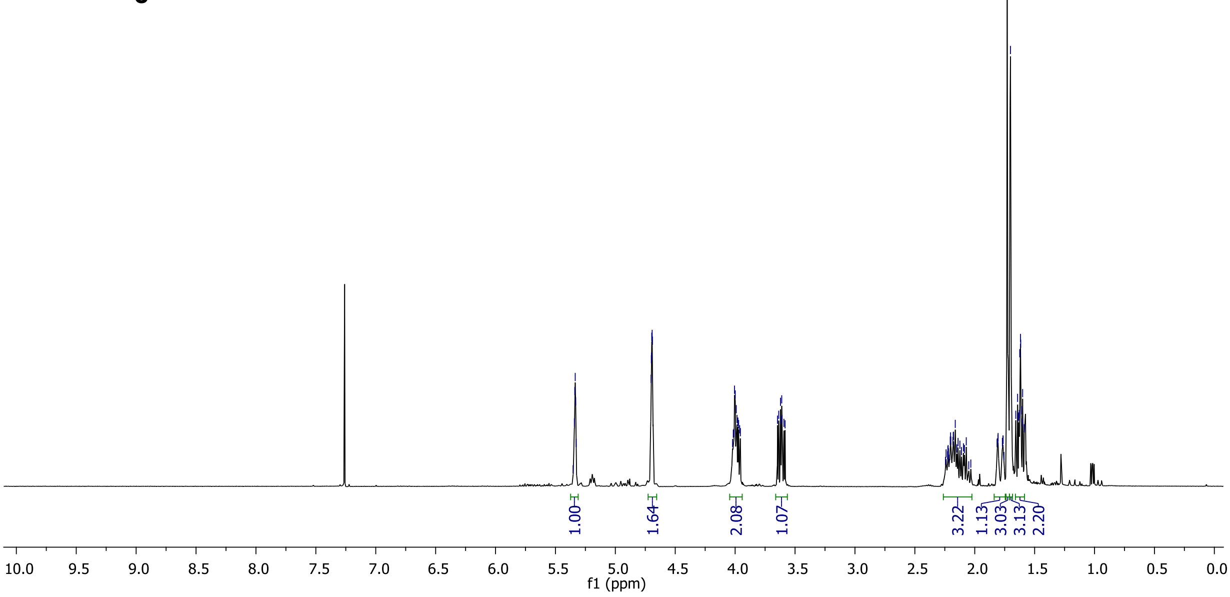
5.34
5.34
5.33
5.33
5.33
4.70
4.70
4.70
4.70
4.69
4.69
4.02
4.02
4.01
4.01
4.00
3.99
3.98
3.98
3.97
3.96
3.96
3.65
3.64
3.62
3.62
3.61
3.61
3.59
3.58
2.24
2.23
2.22
2.22
2.22
2.20
2.20
2.20
2.18
2.18
2.18
2.16
2.15
2.14
2.12
2.11
2.09
2.09
2.07
1.81
1.81
1.81
1.81
1.77
1.77
1.76
1.76
1.73
1.70
1.66
1.64
1.64
1.63
1.63
1.62
1.62
1.62
1.61
1.60
1.60
1.59
1.00
1.64
2.08
1.07
3.22
1.13
3.03
3.13
2.20
10.0 9.5 9.0 8.5 8.0 7.5 7.0 6.5 6.0 5.5 5.0 4.5 4.0 3.5 3.0 2.5 2.0 1.5 1.0 0.5 0.0
f1 (ppm)

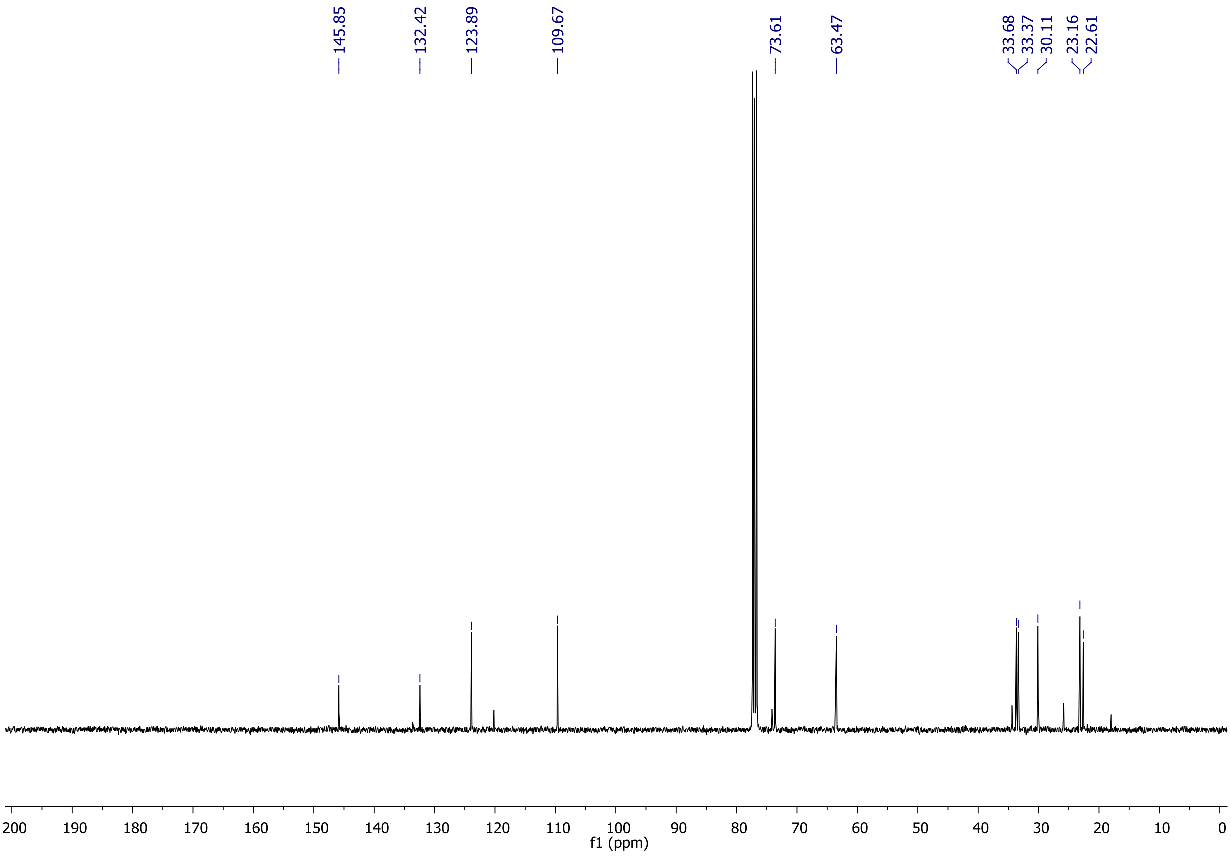
145.85
132.42
123.89
109.67
73.61
63.47
33.68
33.37
30.11
23.16
22.61
200 190 180 170 160 150 140 130 120 110 100 90 80 70 60 50 40 30 20 10 0
f1 (ppm)

Tetrahydroisobenzofurane:

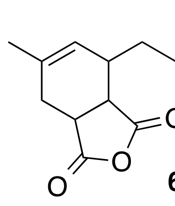

6a

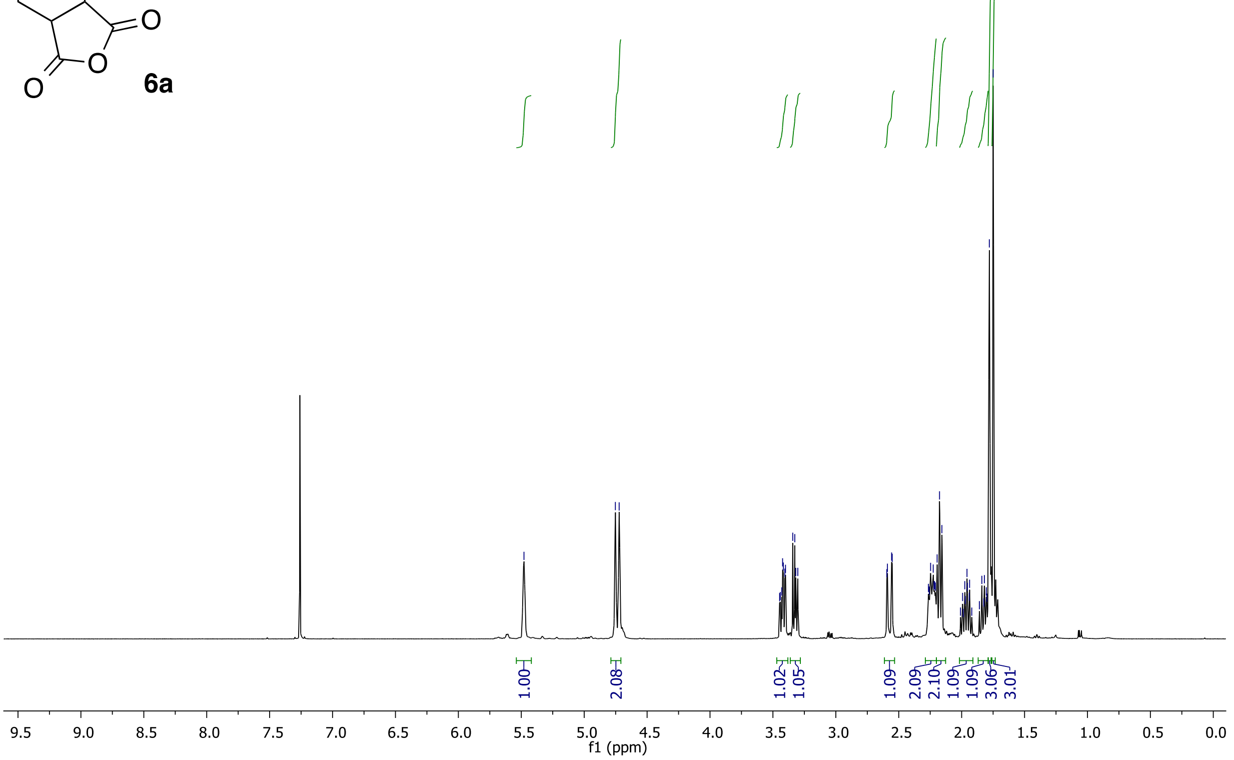

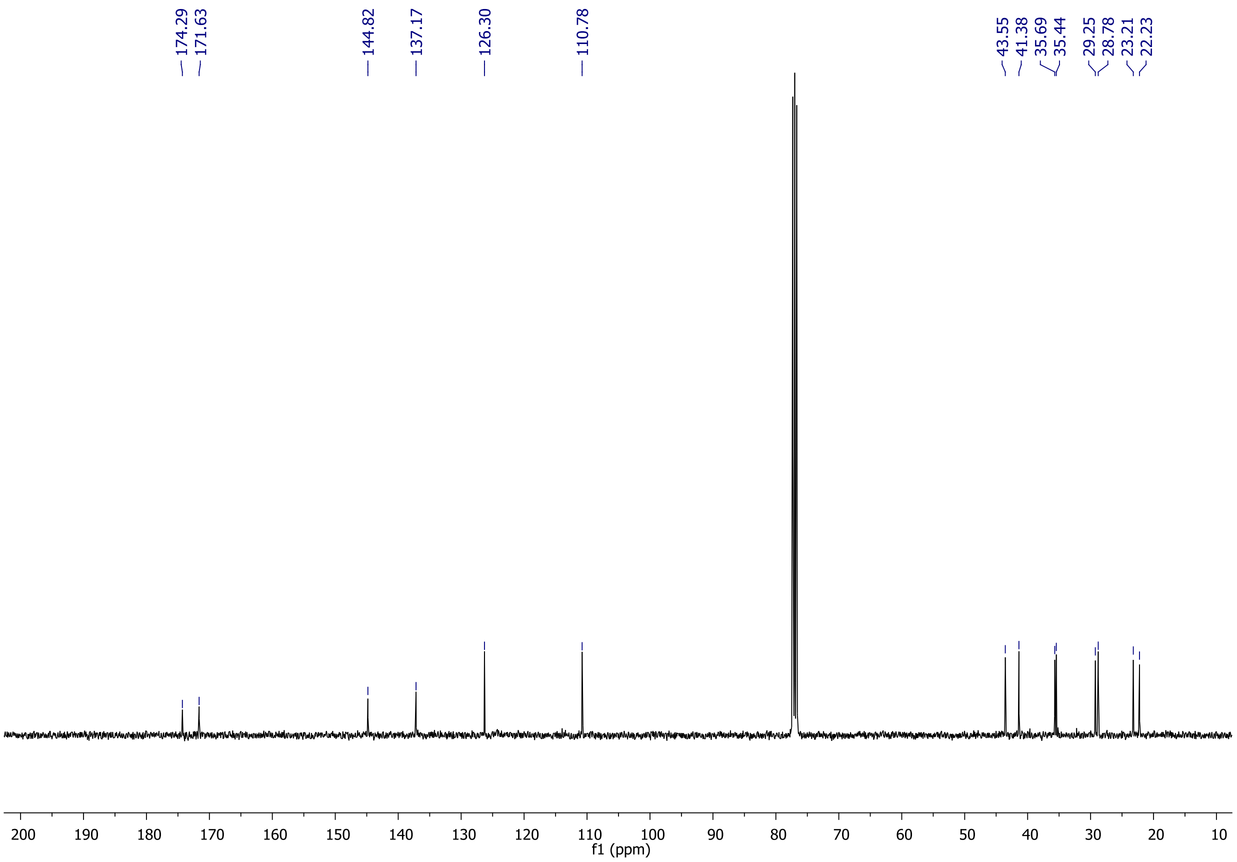

***N*-Phenyltetrahydroisoindole:**

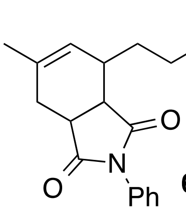

6b

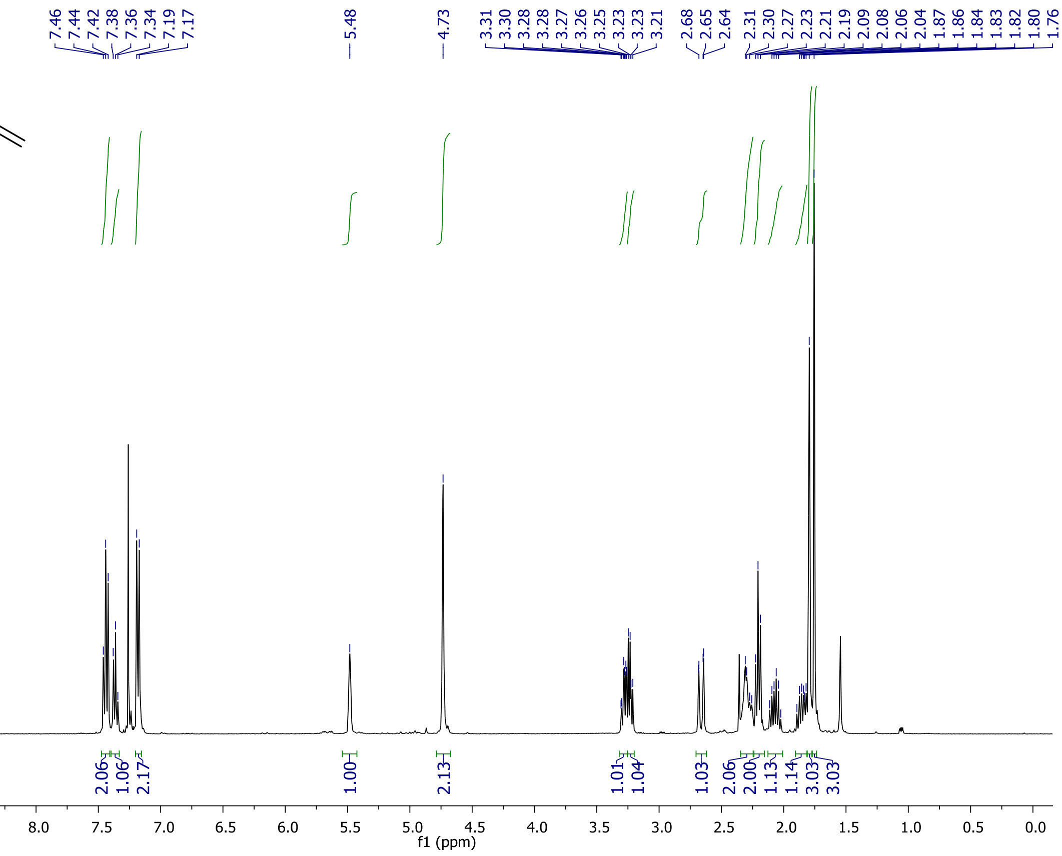

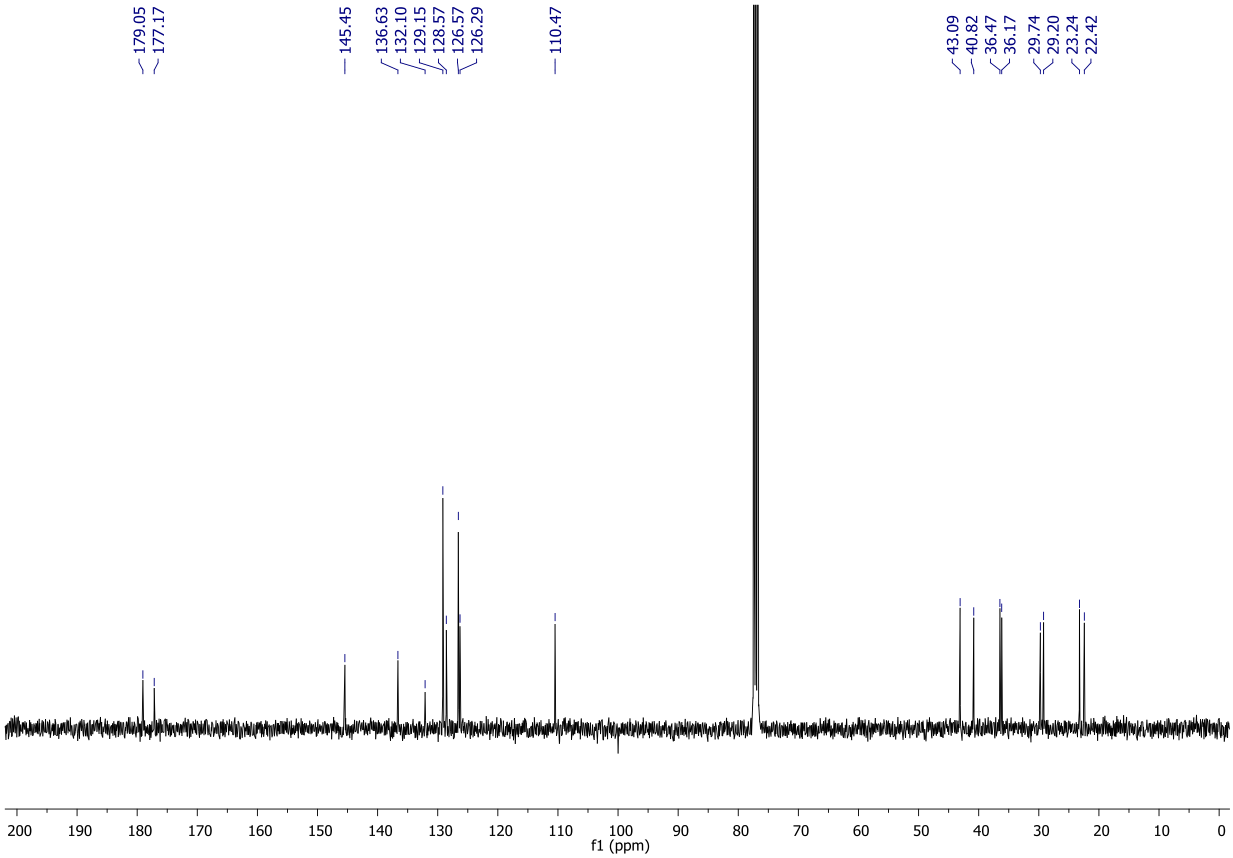

***N*-Methyltetrahydroisoindole:**

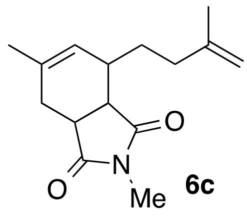

6c

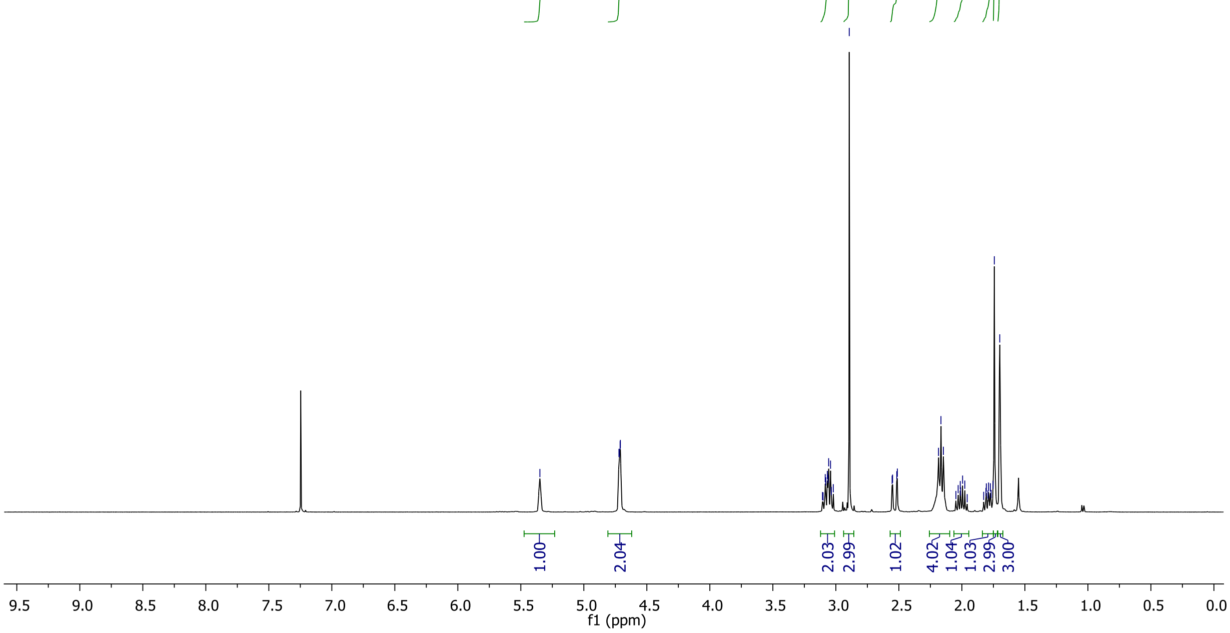

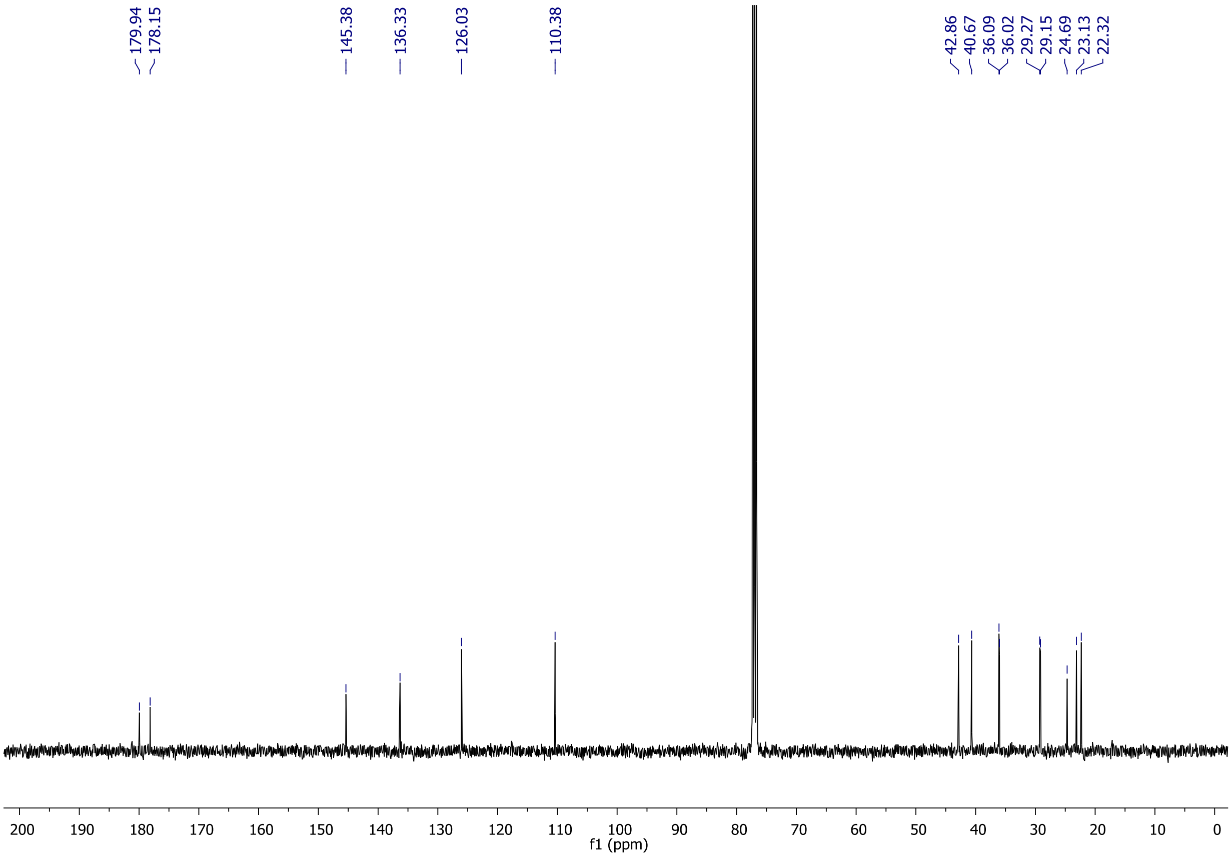

Pyrrolidine:

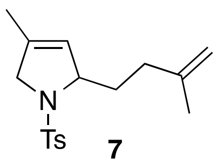

7

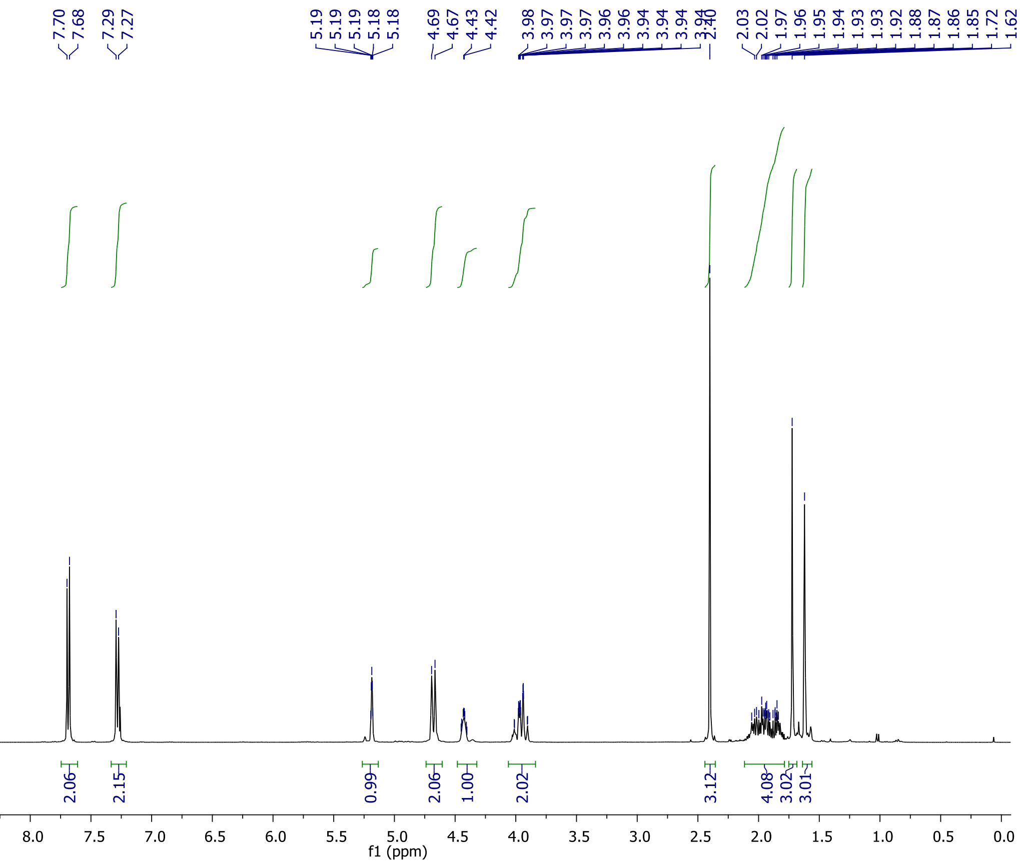

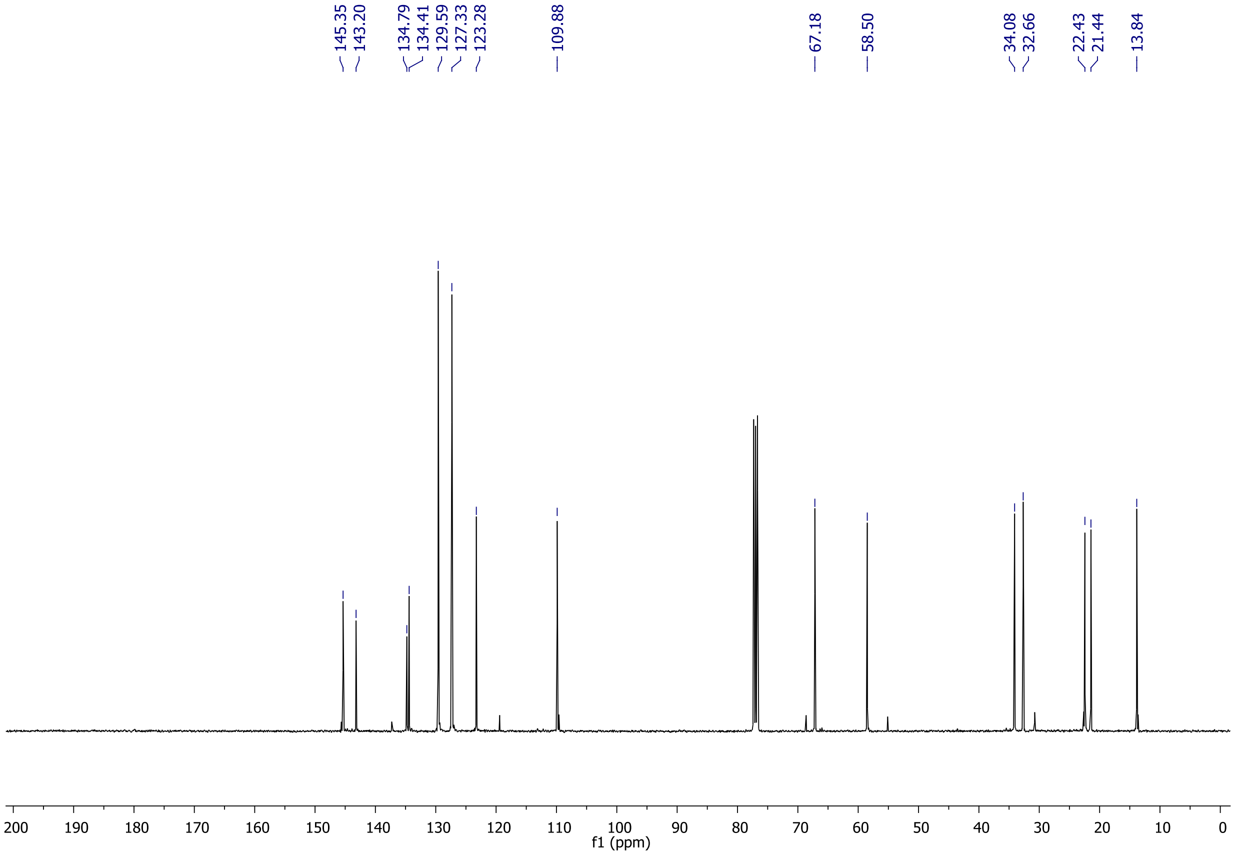

2. GC–FID analysis:

Full chromatogram [standard: decanol, 7.185 min; 2-TT, 5.142 min]

Data Name : C:\GCsolution\Data\Project1\ked dimer THF 3_12042017_1.gcd
Method Name : C:\GCsolution\Data\Project1\Method 1.gcm

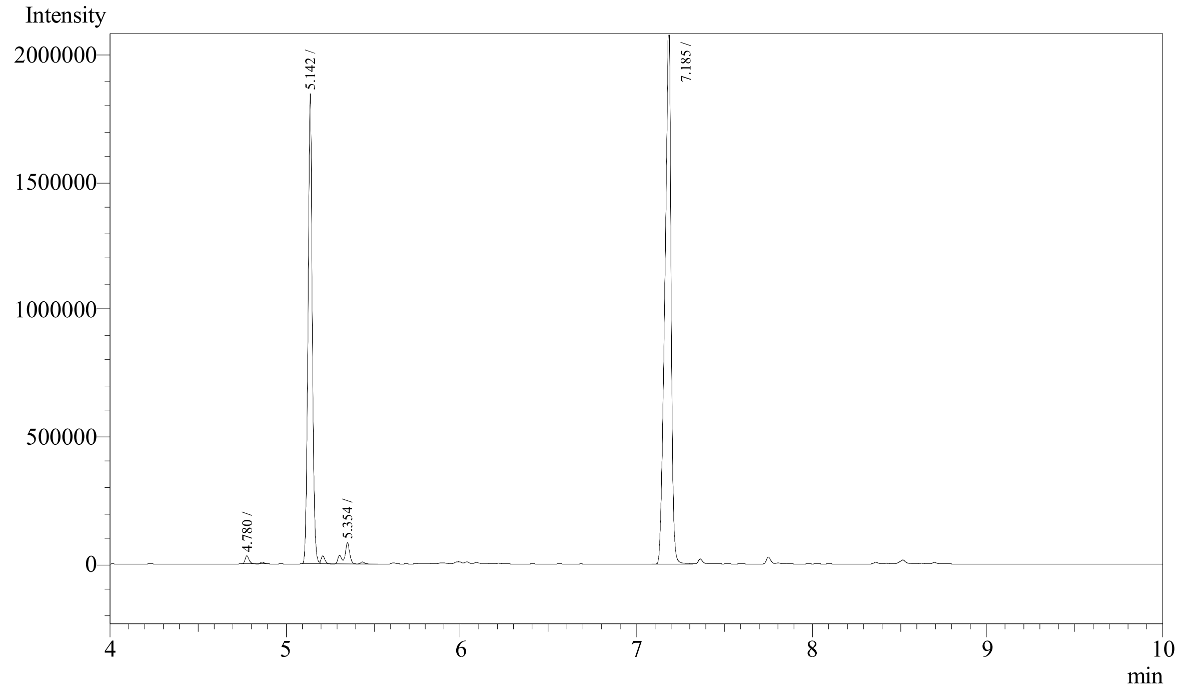

Peak#	Ret.Time	Area	Height	Conc.	Unit	Mark	ID#	Cmpd Name
1	4.780	61293	31678	0.712				
2	5.142	3138420	1797450	36.480				
3	5.354	251007	83015	2.918				
4	7.185	5152360	2132080	59.890				
Total		8603080	4044223					

Zoomed chromatogram (monoterpene species)

Chromatogram ked dimer THF 3 C:\GCsolution\Data\Project1\ked dimer THF 3_12042017_1.gcd - Channel 1

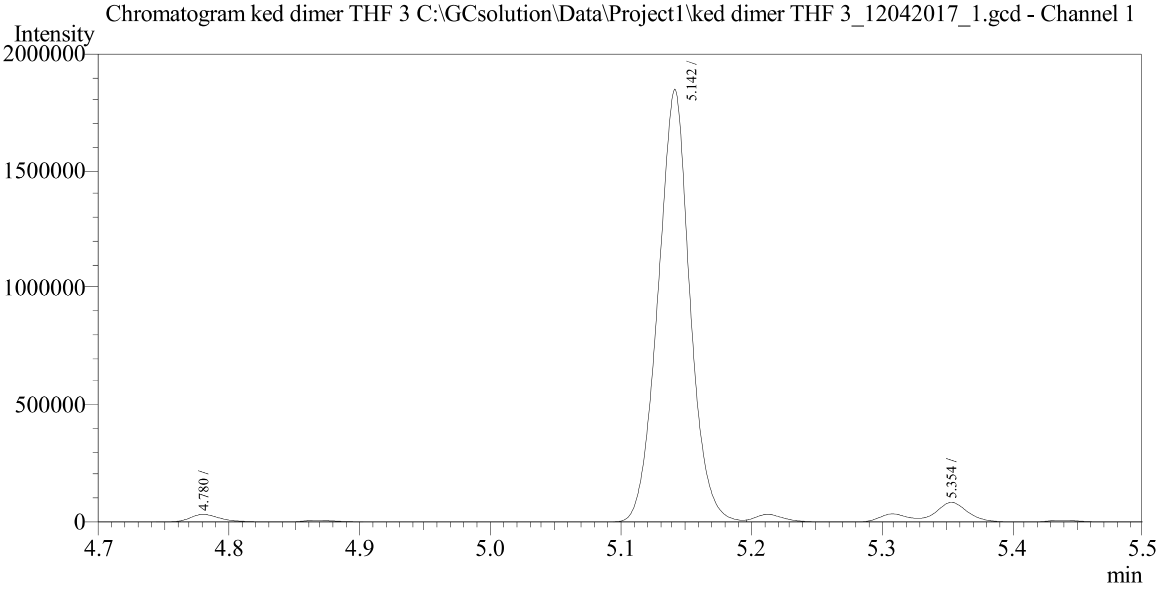

Supplement: File 1 — 1H NMR and 13C NMR spectra collection of the products and GC–FID analysis of the isoprene dimer’s mixture. [file Beilstein_J_Org_Chem-13-1807-s001.pdf]
